# Treatment of Empyema in Children

**Published:** 1868-05

**Authors:** 


### Treatment of Empyema in Children.

Dr. Thomas Hillier, F. R. C. P., Physician to the Hospital for Sick Children and to the Skin Department of University College Hospital, writes upon the subject of empyema in children to the *British Medical Journal* for August 3d, 1867, p. 80:

Pleurisy is a much commoner disease of children than would be supposed from reading the ordinary treatises on children's diseases. Its presence is often overlooked; the symptoms are referred to "infantile remittent," to "congestions of the brain," or to "disordered liver," and, if it become chronic, to phthisis or atrophy. Pain is either not complained of at all, or else it is referred to the abdomen, or shows itself in a general tenderness of the body; hence the chest is not examined. When a physical examination of the thorax is made, from the frequent absence of friction and the presence of bronchial breathing, with the difficulty of getting much information from the voice in young children, the case is often mistaken for inflammation of the lung or tuburcular consolidation.

Idiopathic pleurisy is not at all rare in children; pleurisy, secondary to tubercle, to pneumonia, or to real disease, is equally common, or even more so. Pleuritic effusion in children, even when idiopathic, has a great tendency to become purulent at a very early period. When pus exists in the pleura, as a general rule, the sooner recourse is had to paracentesis the better. Even when the effusion is not purulent, if the pleura is much distended, and dyspnœa is increasing, in spite of diuretics and counter-irritation, it is unadvisable to postpone the operation. If the empyema be of very long standing, and the dyspnœa be not great, and rather on the decrease, it is better to leave the case to nature; and the purulent collection will probably excite no irritation, and its fluid portion may be absorbed. In one case of this kind, in a boy five years old, where the disease was of three years' standing, I operated with the patient's chest under water. The lung, which had been long collapsed, was atrophied; a communication was established between the bronchi and the pleural cavity; this caused decomposition of the contents of the pleura, and the patient died in a fortnight of irritative fever.

Of seventeen cases of empyema of which I have notes, in six there were spontaneous openings or pointing of matter requiring operation; of these, five recovered with permanent fistula; the other died, after fourteen months, with necrosis of the sternum and amyloid degeneration of the viscera. In eleven cases, paracentesis was performed; of these, six recovered completely, one had a permanent fistula, and four died. Of the four fatal cases, one was a child only five months old, who died of collapse of the lung; one was twelve months old, and died of pneumonia; one was the child whose case I referred to just now; and another died of septicæmia, whose case will be reported presently.

I believe it is of great importance to prevent the entrance of air into the pleura. I once put the patient into a warm bath, and opened the chest under water. The result in this case has been above mentioned, but was not due to the mode of operation. In some cases I have evacuated the contents of the chest by means of a long, narrow, elastic tube, whose free end opened under water. Unless air is admitted, and if the lung does not expand very readily, the contents of the cavity are but partially evacuated. To promote more complete evacuation, the employment of an exhausting syringe, as recommended by Dr. Bowditch (*American Journal of Medical Science*, vol. xx, October, 1850, p. 325; and Gairdner's *Clinical Medicine*, pp. 380 and 718,) is of great service.

Of the last six cases in my wards at the Children's Hospital, in which this syringe has been used, five recovered, and only one died. This was a child, twelve months old, who died of pneumonia. He had been previously leeched on his head at a London Hospital, and, when admitted, was extremely anæmic and exhausted. These results are probably more favorable than one can usually expect to meet with. Of Bowditch's cases, in twenty-six the fluid was serous, and twenty-one recovered; in twenty-four it was purulent—of these, eight recovered, nine were relieved, and seven died.

Dr. Hillier then gives a detailed account of his successful cases and comes to the

*Conclusions.*—Paracentesis thoracis is not a dangerous operation in children; if performed early, the chances of a favorable result are very great. The cavity should be evacuated as completely as possible without the admission of air, and then closed. If the contents of the cavity become fetid, a counter-opening should be made, and a drainage-tube introduced, as recommended by Chassaignac and Dr. Goodfellow. Injections of iodine tend to diminish fetor, but have no effect in promoting the closure of the cavity. Change of air, nutritious food and tonics, are the main agents in favoring recovery. Very great deformity of the chest from contraction after pleurisy in children may be completely removed if the lung have not been too long disabled by compression.—*Half-Yearly Compendium.*

